# Quality of CKD Care: Don’t Take the Neighborhood Out of It Yet

**DOI:** 10.1016/j.xkme.2021.06.003

**Published:** 2021-06-30

**Authors:** Jessica L. Harding, Laura C. Plantinga

**Affiliations:** 1 Department of Medicine, School of Medicine, Emory University, Atlanta, Georgia; 2 Department of Surgery, School of Medicine, Emory University, Atlanta, Georgia; 3 Department of Epidemiology, Rollins School of Public Health, Emory University, Atlanta, Georgia

Related article, p. 515

Chronic kidney disease (CKD) is a major public health problem, with an estimated U.S. adult prevalence of 15%.^1^ Moreover, 9 in 10 adults do not know they have CKD,^1^ and quality of care remains suboptimal: for example, from 2013 to 2016, only 43% of U.S. adults with known CKD and diabetes received recommended medical treatment with angiotensin-converting enzyme inhibitors (ACEis) or angiotensin II receptor blockers (ARBs).^2^ The burden of CKD, including suboptimal CKD care, is not equally distributed among populations, with evidence of substantial inequalities across individual socioeconomic status (SES) for a variety of CKD outcomes, including incidence,^3^ disease progression,^4^ CKD awareness,^5^ nephrology care,^6^ and kidney transplant access.^7^ However, health status is influenced by individual attributes as well as the attributes of the environments in which we live, learn, work, pray, and play. Neighborhood context, for example, encompasses the social, economic, and physical features of the residential community and can impact an individual's health, not only by affecting access to health-promoting resources and influencing health-related behaviors,^8^ but also by affecting access to high-quality clinical care.

In this issue of *Kidney Medicine*, Ghazi et al^9^ explore the association between neighborhood-level SES and quality of CKD care using electronic health record (EHR) data from 7 counties in the Minneapolis/St Paul metropolitan area of Minnesota. Patients were included in the study if they were aged ≥18 years; had at least 1 primary care physician visit between July 1, 2017 and December 31, 2018; had a geocoded home address in the metropolitan area; and had at least 1 measure of outpatient creatinine during this time period. Patients with CKD were defined as having an eGFR <60 mL/min/1.73 m^2^, estimated using the CKD-EPI equation.^10^ The 3 measures of neighborhood-level SES, identified from the American Community Survey (2008-2012) and using census tracts, were wealth (median value of owner-occupied housing units), education (percentage of residents >25 years with a bachelor’s degree or higher), and income (median household income), all categorized into quartiles. The 3 measures of CKD care, based on the Healthy People 2020^5^ objectives, were (1) prescription of ACEi/ARB by providers among those with hypertension and CKD (stage 3 or higher, or stage 1 or 2 with urine albumin-to-creatinine ratio [UACR] >300 mg/day) and no contraindications for ACEi/ARB; (2) UACR measurement among people with CKD; and (3) identification of CKD in EHR data in people with laboratory-measured CKD. The authors used regression models to analyze associations between neighborhood SES and CKD care, including a random intercept for census tract level, and adjusted for patient-level age, sex, race, obesity, smoking, insurance status, and comorbidities (cardiovascular disease, stroke, cancer, hyperlipidemia, and diabetes).

Overall, in this sample of ∼16,000-25,000 CKD patients (depending on outcome), Ghazi et al^9^ reported low-to-moderate-quality CKD care. The percentages of CKD patients meeting ACEi/ARB prescription adherence, UACR measurement performance, and CKD identification in EHR were 65%, 27%, and 55%, respectively. Similar estimates have been shown in other studies but vary by underlying population.^5^^,^^11^^,^^12^ Perhaps surprisingly, this study showed that living in a neighborhood with the lowest SES (first quartile), compared to living in a neighborhood with the highest SES (fourth quartile), was not associated with differences in quality of CKD care, after adjusting for demographics and clinical characteristics, regardless of the measure of neighborhood SES or CKD care.^9^

This finding is in contrast to the broader literature, which suggests, in general, that neighborhood SES is related to the risk of incident end-stage kidney disease (ESKD),^13^ access to kidney transplant,^14^ and ESKD mortality.^15^ However, the results are consistent with studies that show no association between neighborhood SES and outcomes among populations with earlier stages of CKD (ie, prior to ESKD).^8^ Previous studies of neighborhood-level poverty and pre-ESKD quality of care also showed mixed findings.^16^^,^^17^ Taken together, it is possible that this study reflects an underlying true lack of association: namely, that neighborhood-level SES is simply not associated with quality of CKD care. However, there are also several alternate hypotheses for these findings, which could be explored in future studies.

**Figure 1:**
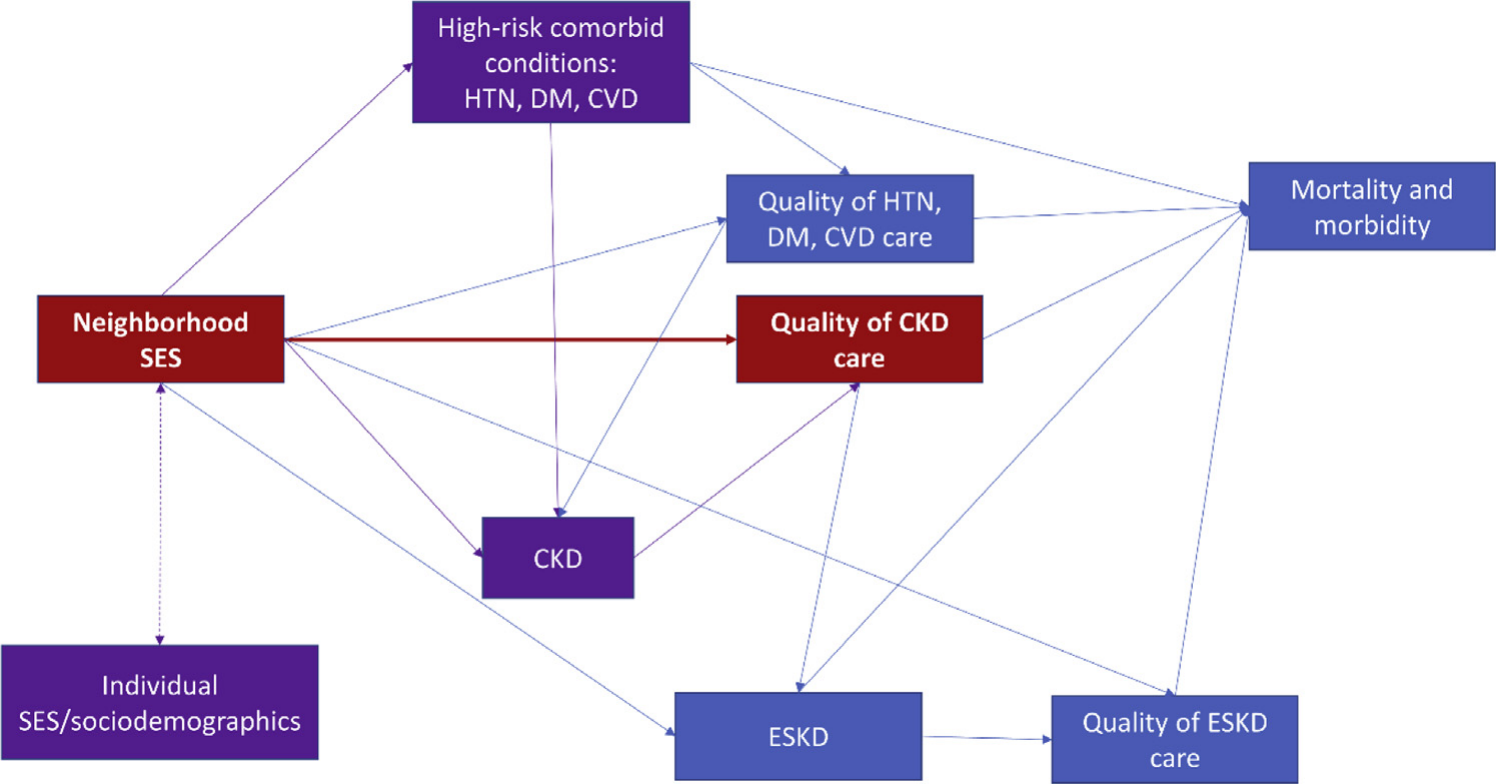
Conceptual model of the association of multiple factors related to neighborhood socioeconomic status and quality of chronic disease care. Red boxes/arrows, associations examined in Ghazi et al^9^; purple boxes/arrows, factors at least partially controlled (through exclusion or statistical adjustment) in Ghazi et al^9^; and blue, other related factors and outcomes. Abbreviations: CKD, chronic kidney disease; CVD, cardiovascular disease; DM, diabetes mellitus; ESKD, end-stage kidney disease; HTN, hypertension; SES, socioeconomic status.

First, associations between neighborhood-level SES and CKD care may be better elucidated in other populations. In this study, the denominator is a nongeneralizable population in a single region seeking primary health care with at least 1 measurement of creatinine. Therefore, it is possible that those in the lowest quartile of SES in the current study do not appropriately represent the true lowest quartile of SES in the target population, owing to decreased likelihood of accessing primary care among lower SES populations.^18^ Further, the definition of CKD for population inclusion was determined from just 1 measurement of creatinine, which is likely to inflate the true number of people with CKD. It is also possible that the impact of neighborhood-level SES occurs upstream of CKD (Fig 1). For example, in this study, the population are those that already have CKD and are accessing primary care, regardless of neighborhood SES. It is possible that neighborhood SES may have a greater impact among those considered at high risk for CKD, ie. those with diabetes or hypertension, in preventing progression to CKD, compared to the impact among those who have already developed CKD.

Second, the markers of neighborhood-level SES used in this study may not capture features of the neighborhood that are most important for optimal CKD care. For example, as a measure of wealth, Ghazi et al^9^ use median value of owner-occupied housing units, which may not reflect the proportion of people in that census tract who live in owner-occupied housing. In a city like San Francisco, for example, the median house value is US$1.7 million,^19^ yet almost half (∼45%)^20^ of households in this area are rented and individuals in these households may not experience this wealth. Measures of racial segregation and/or economic inequality, rather than the SES of the immediate neighborhood, may better capture the resources and constraints important for the provision of CKD care^8^; for example, racial segregation has been associated with ESKD mortality among Black dialysis patients.^15^ In addition, the current study examines 3 independent measures of neighborhood-level SES, but does not consider combinations of these measures (eg, an area deprivation index), the complex interplay between features of the neighborhood context, or the interaction of neighborhood factors with individual SES and sociodemographic factors (Fig 1), all of which may be important for understanding the correlates of quality of CKD care.

Last, it is possible that the exclusions and adjustments in Ghazi et al^9^ partially mask the associations of interest. While it was necessary to limit to those with CKD to examine the measures chosen by the authors, CKD itself may be a result of the quality of care at phases upstream of CKD (Fig 1). Quality of care for risk factors related to the development of CKD may be more strongly affected by neighborhood SES,^8^ and so the exclusion of the high-risk population without CKD may have removed a substantial contribution to the causal pathway being examined. Furthermore, the variables chosen for adjustment include both high-risk comorbid conditions and individual sociodemographic variables (Fig 1), which may act as mediators or effect modifiers instead of or in addition to confounders, further lessening the observed overall association (although it should be noted that unadjusted associations were also null, or close to). Future analyses could examine additional covariates (including life-course individual and neighborhood SES), explore alternate pathways, and consider mediation analyses and potentially stratified analyses (in the presence of effect modification) to tease apart these complex effects.

The current study by Ghazi et al^9^ adds to the growing body of evidence that quality of CKD care is suboptimal overall and presents an opportunity for improvement. While the authors did not find associations of neighborhood SES with quality of CKD care, it is important to note that no single study can address all the complexities involved in considering the neighborhood context in individual and population health; in fact, this novel study can be considered preliminary and hypothesis-generating. Future studies on neighborhood factors and quality of CKD care can address these hypotheses by examining associations in diverse populations in terms of geography, culture, and access to care; incorporating various measures of neighborhood SES, including measures of racial segregation and economic inequality and combined deprivation indices; and using innovative approaches to examine the complex relationships between quality of care related to risk factors, CKD, and ESKD as well as between neighborhood and individual SES. Together, such studies will contribute to potential future interventions at the health care and policy levels to improve quality of CKD care and, ultimately, patient outcomes.
